# Comparison between four different suture configurations for rotator cuff repair: a biomechanical animal study

**DOI:** 10.1051/sicotj/2024038

**Published:** 2024-10-07

**Authors:** Yahia Haroun, Mohamed H. Sobhy, Hany A. Khater, Ahmad H. Khater

**Affiliations:** 1 Orthopedic Surgery, Faculty of Medicine, Ain Shams University 38 Abbassia, next to the Al-Nour Mosque 11566 Cairo Egypt; 2 Orthopedic Surgery, Ain Shams University 38 Abbassia, next to the Al-Nour Mosque 11566 Cairo Egypt; 3 Mechanical Power Engineering, Faculty of Engineering, Cairo University 12613 Giza Egypt

**Keywords:** Mason Allen stitch, Rotator cuff tear, Biomechanical

## Abstract

*Introduction*: The arthroscopic repair of a massive rotator cuff tear could be surgically challenging. There is a continuous argument regarding the best surgical technique and suture configuration used to treat massive rotator cuff tears. The purpose of this study was to assess the in vitro strength of the new double Mason-Allen suture and compare it to the commonly used other suture configurations. *Methods*: Twenty-five fresh sheep shoulders were randomly divided into five equal groups. Each group had their infraspinatus tendons cut and repaired with one of five suture configurations (simple, horizontal mattress, Mason-Allen, modified Mason-Allen, and double Mason-Allen) using Arthrex^®^ 1.3 mm suture tape. The specimens were fixed to the test apparatus through their scapulae and hung with the repair tape to Sartorius^®^ precision balance with sequential load increments till failure. The load to failure was measured for each of the five suture configurations. *Results*: Study data found the double Mason-Allen configuration to have the highest mean load to failure 423.30 ± 23.05 (Newtons), followed by modified Mason-Allen, Mason-Allen, horizontal mattress, and simple suture respectively. *Conclusion*: The double Mason-Allen repair configuration has the highest load to failure compared to the other known suture configuration to repair rotator cuff tears.

## Introduction

The arthroscopic repair of a massive rotator cuff tear could be surgically challenging with a high incidence of retear [1–3], and complete repair is complicated because of tendon retraction, muscle atrophy, and fatty degeneration [4]. The suture technique for grasping the torn tendon should provide high initial fixation strength, to prevent gap formation and maintain mechanical stability until healing has occurred [5, 6]. The Mason-Allen stitch (MAS) is known to be the strongest suture configuration [7]. Habermeyer et al. modified this configuration to be done arthroscopically, known as the modified Mason-Allen stitch (MMAS) [8]. A continuous argument regarding the best surgical technique and suture configuration to treat massive rotator cuff tears remains a surgical challenge [8]. Suture cutting out of the tendon and retear medial to tendon muscle junction are frequently reported issues that often require complex surgical techniques and additional anchors, which can be disadvantageous for surgeons and increase the economic burden for the patient [9–11]. Despite recent advancements in arthroscopic rotator cuff repair, the outcomes remain unsatisfactory, with no consensus reached [11, 12]. Therefore, innovations in this respect are necessary [11]. So far, limited studies have shown the comparative efficacy of different suture techniques [11]. Accordingly, surgeons base their choices on personal experience [11]. We described the double Mason-Allen configuration; its strength has not been previously reported. The primary purpose of our study is to measure the load to failure of the newly proposed double Mason-Allen suture configuration. The secondary purpose is to compare it to the commonly used suture configurations. We hypothesize that the double Mason-Allen repair configuration has the highest load to failure.

## Materials and methods

This study was conducted on twenty-five male sheep infraspinatus tendons aged between 13 and 24 months. The specimens were obtained as whole sheep shoulders and used fresh without freezing. The soft tissue was dissected off the shoulder bony skeleton to expose the infraspinatus tendon and muscle. The infraspinatus tendon was thoroughly examined for any abnormalities or tears, then the tendon was cut from its humeral insertion, mimicking a full-thickness rotator cuff tear.

The specimens were randomly allocated to one of five groups (5 shoulders per group) in the following configurations:

Group 1 was allocated to the simple suture configuration (the suture tape passing only once from the articular side to the bursal side) [13].

Group 2 was allocated to the horizontal Mattress suture (the two limbs of the suture tape passed from the articular side to the bursal side) [13].

Group 3 was allocated to MMAS described by Habermeyer 2003 (two suture tapes, one in mattress fashion 1 cm from the torn tendon edge, and the second was passed in a simple fashion medial and between the mattress sutures) [6].

Group 4 was allocated to MAS described by Gerber et al. 1994 (one suture tape passed three times in the tendon substance, the first passage was from the articular to the bursal side 1 cm from the torn tendon edge, the second passage was from the bursal to the articular side about 1 cm from the first, finally, the last passage was from the articular to the bursal side between the first two passages and medial to them) [13].

**Figure 1:**
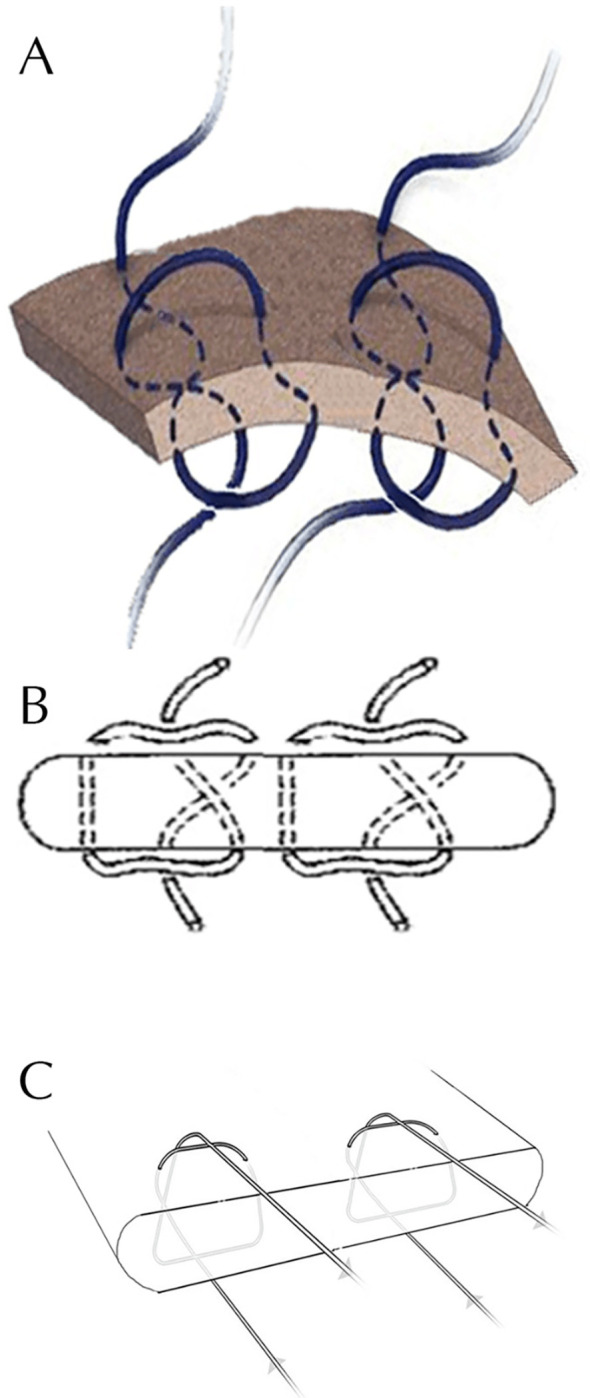
A diagrammatic representation of the double Mason-Allen suture configuration. A, B and C reveal different diagrammatic representations of the double Mason-Allen suture.

Group 5 was allocated to double MAS suture (the MAS configuration was done twice on the same tendon specimen, where the 2 sutures were placed at a 5 mm distance from each other) (Figure 1).

All the suture passages were done by Arthrex Scorpion^®^ device using 1.3 mm suture tape (Arthrex, Naples, FL, USA) through the torn tendons.

Every specimen was then fixed to the test apparatus through its scapula. The suture tape-free end was hung to Sartorius^®^ precision balance model QS64B (Sartorius AG, Germany). Reference Line Weights according to R111-1 OIML Class M1 was used to increase the weight on the suture tape in a controlled manner of 100 g until failure. Weight and load to failure were recorded for analysis for each specimen.

### Statistical analysis of data

Collected data were stored in Microsoft^®^ Excel^®^ for Microsoft 365 MSO (Version 2302 Build 16.0.16130.20848) 64-bit and analyzed using the statistical package for social sciences, version 20.0 (SPSS Inc., Chicago, IL, USA). Quantitative data were expressed as mean ± standard deviation (SD). The ANOVA test was conducted, followed by post hoc analysis to determine differences between the groups in relation to load-to-failure responses. The confidence interval was set to 95% and the margin of error accepted was set to 5%. So, the *P* value was considered significant as the following: Probability (*P* value)

- *P* value less than 0.05 was considered significant.
- *P* value less than 0.001 was considered highly significant.
- *P* value greater than 0.05 was considered insignificant.

## Results

Comparing the weights and loads to failure of the five suture configurations, the double Mason-Allen stitch had the highest mean load to failure of 423.30 ± 23.05 (Newtons) followed by the Modified Mason-Allen stitch. The difference between the two was statistically significant with a *p*-value < 0.05.

The results indicate that the proposed double Mason-Allen configuration has a higher rupture force by about 265% compared to the MMAS, which is a significant difference. Also, the percentage increase in rupture force for the double Mason-Allen was higher than all the other tested suture configurations.

The mean load causing suture failure between the five groups was statistically different, with a *p-value* < 0.001. The five groups can be arranged in decreasing order of mean load to failure as follows: double Mason-Allen, MMAS, MAS, horizontal mattress, and simple suture respectively (Table 1). Table 2 summarizes the pros and cons of each technique.

**Table 1:** Results obtained for the required load to rupture the tested suture configuration, including the new proposed double Mason-Allen.

Tests	Actual test results			
	Rupture weight	Load to failure	Results	Error due to using 100 g weight steps
			Mean load to failure ± standard deviation	
	(g)	(N)	(N)	(%)
**1) Simple**				
Minimum	2,900	28.45	33.84 ± 5.40	3.5
Maximum	4,000	39.24		2.5
**2) Mattress**				
Minimum	5,200	51.01	66.22 ± 15.21	1.9
Maximum	8,300	81.42		1.2
**3) Mason-Allen**				
Minimum	14,000	137.34	145.68 ± 8.34	0.7
Maximum	15,700	154.02		0.6
**4) Modified Mason-Allen**				
Minimum	15,200	149.11	159.90 ± 10.79	0.7
Maximum	17,400	170.69		0.6
**5) Double Mason-Allen**				
Minimum	40,800	400.25	423.30 ± 23.05	0.2
Maximum	45,500	446.36		0.2

**Table 2:** Summary of the pros and cons of the tested suture configurations.

	Pros	Cons
Simple	– Easy and commonly used	– Inferior mechanical strength
	– No tissue strangulation	– Fail to reestablish the normal footprint
Horizontal Mattress	– Easy and commonly used	– Tissue strangulation
Modified Mason-Allen	– Higher load to failure compared to simple, horizontal mattress and Mason-Allen	– Requires skilled surgical technique
	– Less strangulation	
Mason-Allen	– Higher load to failure stitch compared to simple & horizontal mattress	– Requires skilled surgical technique
	– Less strangulation	
Double Mason-Allen	– Highest load to failure compared to above-mentioned configuration	– Requires skilled surgical technique

## Discussion

The optimal surgical technique and suture configuration for the arthroscopic repair of a massive rotator cuff tear is still a topic of debate [8]. This study demonstrated that the double Mason-Allen suture configuration had the highest load to failure, with a mean of 423.30 ± 23.05(Newtons). Conversely, the simple suture was the weakest. This study established that both the double Mason-Allen and modified Mason-Allen sutures had the highest biomechanical properties for the load to failure, suggesting the double MAS to be a better choice for repair configuration. To the best of the author’s knowledge, no studies in literature assessed the double Mason-Allen and compared it to the well-established suturing techniques.

The study had some limitations, such as not investigating other suture configurations that could have possibly shown more strength. Additionally, the challenge of osteoporotic bone and suture anchor pullout could not be tested as the bony insertion was removed. Lastly, fresh tendons were used for repair, which may not accurately replicate the tendons in chronic tears.

It has been demonstrated that the infraspinatus tendon of sheep has characteristics similar to those of the supraspinatus tendon in the human shoulder [14]. This makes it an effective model for studying rotator cuff diseases [14]. Repairing of chronic, massive rotator cuff tears is challenging and has a high rate of re-ruptures [15]. The weakest point of the repair is where the suture meets the tendon. So, achieving a secure tendon fixation is critical until biological healing occurs [15]. Various grasping techniques have been proposed to minimize suture failure and prevent cutting through the tendon [16]. In 2006, Tao and Kaltenbach showed the reproducibility and advantages of the modified Mason-Allen suture configuration and stated that it may improve the healing rates of rotator cuff repair done using a suture anchor [17]. In accordance with our results, in a study on fourteen sheep shoulders, Sileo and colleagues found that the modified Mason-Allen stitch and Mason-Allen stitch yield similar biomechanical features when suture-anchored into bone [18]. In an experimental in-vitro analysis in 2004 Ma and colleagues concluded that the MMAS provides strength with a mean value of 233 ± 40 (Newtons), comparable with that of the MAS with a mean value of 246 ± 40 (Newtons) [19].

For massive, contracted tears, it is often only possible to achieve single-row fixation [16]. Using multiple pairs of Mason-Allen stitches creates additional fixation points, resulting in reduced load per point and increased load to failure that the soft tissue can bear [20].

In our study, suture failure in all the 25 studied specimens occurred at the tendon grip interface, and no suture material tear was noted, this is explained by the very high resistance of the suture material used (suture tape) [21].

## Conclusion

The double Mason-Allen repair configuration has the highest load to failure compared to the other known suture configuration to repair rotator cuff tears.

## Recommendations

We recommend conducting a large-scale in vitro study using the double Mason-Allen suture as a preliminary step before performing surgical cuff repair. Subsequently, the short- and long-term outcomes of the suture on patients should be tested.

## Data Availability

The datasets used and/or analyzed during the current study are available from the corresponding author upon reasonable request.
